# Chronic expanding haematoma in the kidney following long-term hydronephrosis

**DOI:** 10.1093/bjrcr/uaaf069

**Published:** 2025-12-29

**Authors:** Kenta Nishimura, Yasuji Ryu, Natsuki Sugimori, Keisuke Ichimatsu, Takahiko Nakajima, Masatoshi Imamura

**Affiliations:** Department of Radiology, Tonami General Hospital, Tonami, Toyama 939-1395, Japan; Department of Radiology, Tonami General Hospital, Tonami, Toyama 939-1395, Japan; Department of Radiology, Tonami General Hospital, Tonami, Toyama 939-1395, Japan; Department of Radiology, Tonami General Hospital, Tonami, Toyama 939-1395, Japan; Department of Radiology, Tonami General Hospital, Tonami, Toyama 939-1395, Japan; Department of Radiology, Tonami General Hospital, Tonami, Toyama 939-1395, Japan

**Keywords:** computed tomography, magnetic resonance imaging, chronic expanding haematoma, kidney, hydronephrosis

## Abstract

Chronic expanding haematoma is defined as a haematoma that gradually increases in size for ≥1 month. It is mostly related to trauma or surgery and can develop at any location in the body. Herein, we describe a case of a chronic expanding haematoma in the left kidney in a patient with a history of long-term hydronephrosis. The patient was a man in his 70 s who had undergone surgery for seminoma with lymph node metastasis 40 years prior. Following the procedure, he had been in an extended state of hydronephrosis. Computed tomography performed during the follow-up period revealed nodules in his persistently dilated left renal pelvis. A left nephrectomy was performed because the possibility of another malignancy could not be ruled out. Pathological examination revealed that the mass was a haematoma that had originated from the left kidney. To the best of our knowledge, this is the first clinical report in the literature of chronic expanding haematoma in the kidney.

## Clinical presentation

The patient was a man in his 70 s with a history of left testicular seminoma, for which he underwent a left orchidectomy and resection of the accompanying retroperitoneal lymph node metastasis 40 years earlier. Since then, his middle ureter was stenosed, and he had been in an extended state of hydronephrosis. Eleven years prior, he experienced a recurrence of retroperitoneal lymph node metastasis, which subsequently regressed with chemotherapy. Since then, the patient had been followed-up with through yearly hospital visits. Computed tomography (CT) performed during one of these follow-up visits revealed slowly enlarging nodules in his persistently dilated left renal pelvis. Retrospectively, the nodules had been present for more than 10 years; however, they were unnoticed, probably because they resembled debris on simple CT imaging. The patient reported no specific symptoms, and no haematuria was observed.

## Investigations/imaging findings

**Figure 1.**
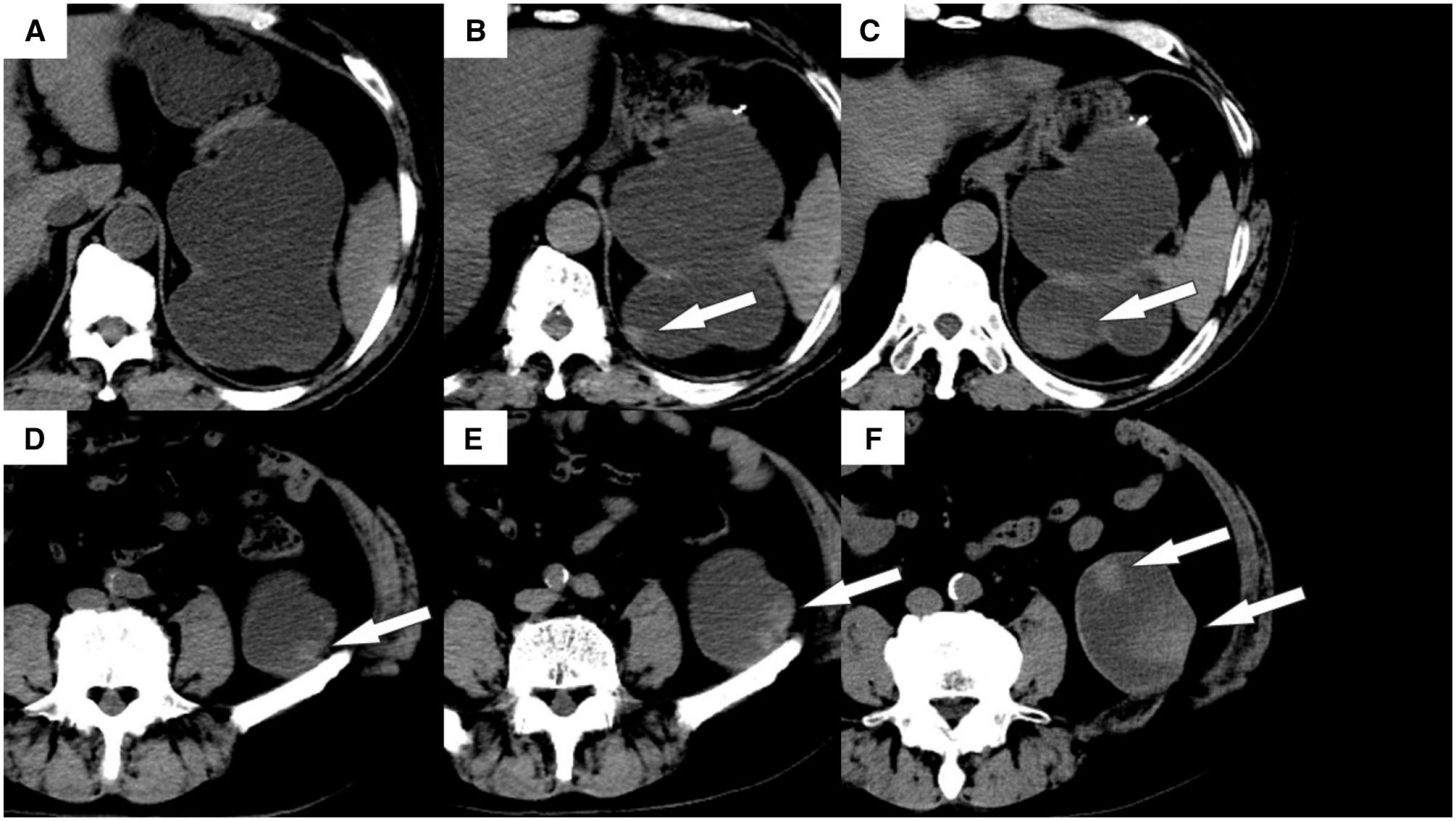
Computed tomography images of the patient’s left kidney. High-density nodules were observed that gradually increased in size over an extended period, ultimately determined to be chronic expanding hematoma (CEHs) (arrows). (A) Ten years before the patient’s presentation at our centre, (B) five years prior, (C) on the day when the CEHs are first noted, (D) ten years prior, (E) five years ago, and (F) on the day when the CEHs are first noted.

**Figure 2.**
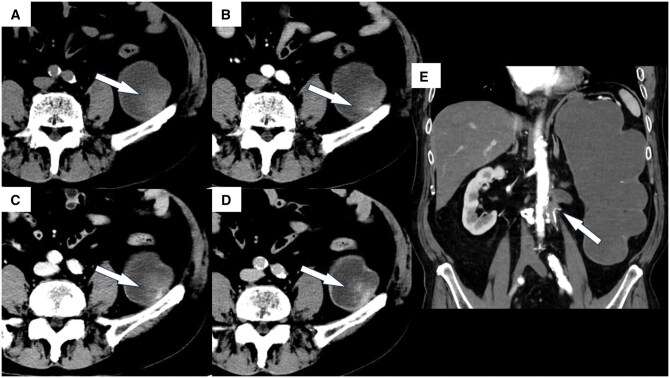
Contrast-enhanced computed tomography images of the patient’s left kidney. (A) Pre-contrast, (B) arterial phase, (C) delayed phase, and (D) excretory phase. Contrast enhancement of up to 40 HU was observed in some parts of the nodules. (E) coronal image of the arterial phase. The arrow shows the obstruction causing long-term hydronephrosis. The curved, high dense structure is presumed to be a suture material from a surgery performed 40 years prior.

**Figure 3.**
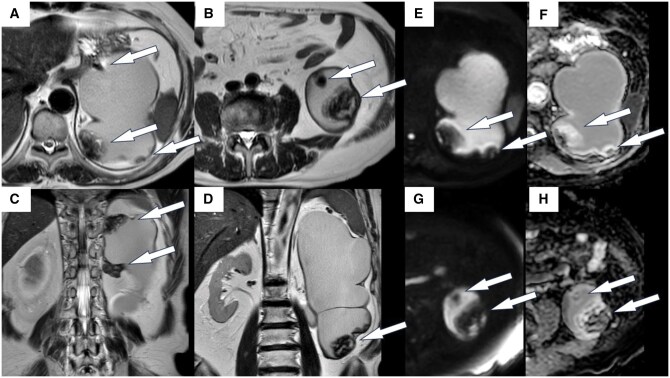
Magnetic resonance images of the patient’s left kidney. This was performed 1 month following the CT scan shown in Figure 1. (A) and (B) Axial views and (C) and (D) coronal views. There were many 10 mm nodules along the wall of the renal pelvis. T2-weighted imaging reveals a mixture of high- and low-intensity areas in the nodules, referred to as a “mosaic sign.” The nodules included capsules. (E) and (G) Diffusion weighted image and (F) and (H) apparent diffusion coefficient map. No evidence of restricted diffusion was observed.

CT showed multiple 10-mm nodules in the dilated left renal pelvis. Each nodule had a higher density than that of the background renal pelvis. These nodules were slowly enlarging in size compared to previous CT findings (Figure 1). They also exhibited contrast-enhancing effects. Magnetic resonance imaging (MRI) revealed a mixed high-to-low signal intensity on T2-weighted imaging, with areas of contrast enhancement similar to those observed on CT (Figures 2 and 3). No other lesions were visible.

## Differential diagnosis

The mosaic sign observed on MRI was suggestive of haematoma; however, the possibility of a malignant renal tumour that also included haemorrhage could not be excluded. The tendency of the lesions to grow in size and exhibit contrast-enhancing effects would have been common to both haematomas and malignant tumours, making a definitive diagnosis based on imaging alone challenging.

## Treatment/outcome/follow-up

As the possibility of renal pelvic cancer could not be ruled out and we considered that a nephrectomy would have had a minimal impact on the patient’s renal function because the left kidney was no longer functional, a left total nephroureterectomy was performed.

**Figure 4.**
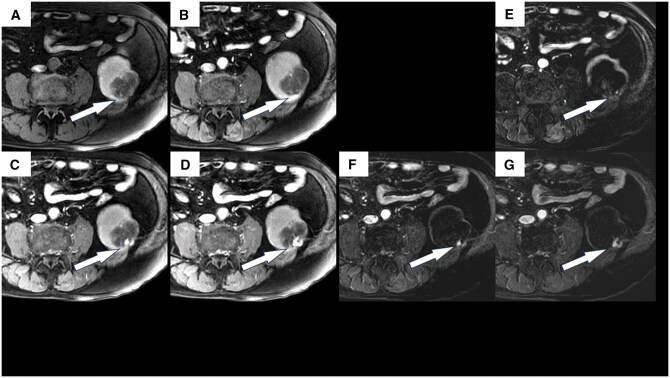
Gadolinium-enhanced dynamic magnetic resonance imaging of the patient’s left kidney. This was performed concurrently with the imaging shown in Figure 3. (A) Pre-contrast, (B) arterial phase, (C) portal venous phase, and (D) delayed phase. Contrast enhancement was observed in some parts of the nodules. (E)–(G) are subtracted images. (E) arterial phase, (F) portal venous phase, and (G) delayed phase.

**Figure 5.**
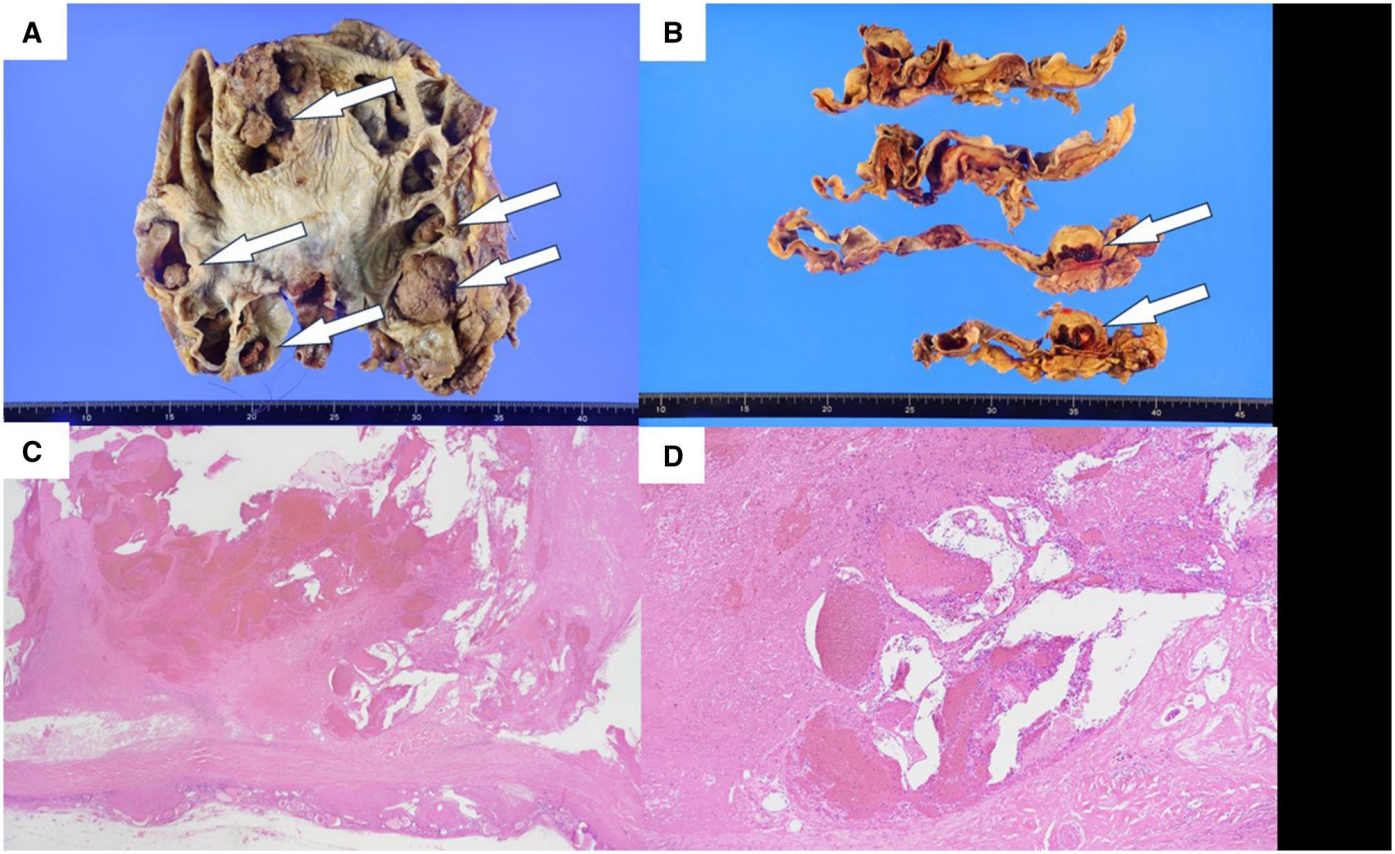
Macroscopic and pathological findings of the left kidney. Macroscopic findings. (A) and (B) Multiple nodules are identified in the left kidney. The nodules were located in the renal column, not in the renal pelvis. Pathological findings. (C) The nodules are determined to consist of haemorrhages. (D) Dilated capillaries are observed.

The nodules, which were believed to be in the dilated renal pelvis during CT and MRI, were located in the left renal column during surgery. Pathologically, it showed dilated capillaries developed around the nodules, indicative of haematomas, with no evidence of cancer (Figures 4 and 5). The patient experienced no recurrence or complications after resecting the nodules.

## Discussion

We recently experienced and successfully treated a case of chronic expanding haematoma (CEH) of the left kidney. CEH was first reported by Reid et al. in 1980,^1^ and is defined as a haematoma that gradually expands over months to years.^2^

The exact mechanism responsible for CEH remains unclear, although it is rarely spontaneous and most commonly observed following various types of injuries, including surgical interventions, seat belt syndrome, and blunt trauma.^3^ It can develop at any location in the body, such as the subcutaneous tissue and muscles of the arm or legs, brain, or thorax, as well as in the abdominal cavity in rare cases.^4^ To the best of our knowledge, no reports of CEH in the kidney have been published.

We initially thought that our patient’s nodule might be a haematoma, although this is a rare occurrence in the kidneys. The mosaic pattern observed on MRI and slight contrast enhancement supported this conclusion; however, we could not rule out the possibility of certain malignant renal tumour types. The nodules gradually increased in size, and contrast enhancement was observed in some areas, which may be present in both haematomas and cancers.

CEH should be diagnosed using various imaging modalities.^3^ Rim enhancement during the arterial phase can be identified via dynamic CT scanning because the haematoma capsule contains granulation tissue with vascular channels.^5^ The MRI signal within the lesion can change over time, indicating temporal changes in haemoglobin levels.^3^ T2-weighted imaging is useful for observing mixed signals, also known as mosaic signs.^6^ There were multiple nodules, which were atypical for renal cell carcinoma.

In this case, the patient’s renal pelvis was dilated until surgery, and the renal pelvis collapsed after the resection. Pathological analysis revealed that the nodules were located in the renal column, not in the renal pelvis.

This patient had a long history of hydronephrosis. Rare cases of renal tumours that occurred following hydronephrosis have been reported.^7^ Long-term hydronephrosis is believed to contribute to tumour development through persistent chronic irritation of the renal pelvis and ureteral mucosa with constant weak inflammatory stimulation of the stagnant urinary tract.^8^

Similar to cases of renal tumours in patients with long-term hydronephrosis, we believe that CEH could grow in a stagnant liquid by taking up small haemorrhage caused by constant weak inflammation. Although the mechanism underlying CEH formation remains unclear, it may involve closed cavities, including hydronephroses or cysts.

To treat CEH, aspiration of the contents of the material or cyst may not prove to be effective due to the remaining unresected fibrous wall. Complete surgical excision, including pseudo-capsule removal, is the optimal treatment for CEH, as aspiration of the fluid content or incomplete excision can lead to either an unconfirmed diagnosis or recurrence.^9^

The nodule in this case was either a haematoma or a malignant tumour—both of which warrant full excision.

## Learning points

- CEH is a haematoma that gradually expands over months to years.
- CEH in the kidney is exceedingly rare.
- CEH should be diagnosed using various imaging modalities, including MRI for detecting mosaic signs.
